# Association Between Financial Incentives and Participant Deception About Study Eligibility

**DOI:** 10.1001/jamanetworkopen.2018.7355

**Published:** 2019-01-25

**Authors:** Holly Fernandez Lynch, Steven Joffe, Harsha Thirumurthy, Dawei Xie, Emily A. Largent

**Affiliations:** 1 Department of Medical Ethics and Health Policy, Perelman School of Medicine, University of Pennsylvania, Philadelphia; 2 Leonard Davis Institute of Health Economics, University of Pennsylvania, Philadelphia; 3 Children’s Hospital of Philadelphia, Pennsylvania; 4 Department of Biostatistics, Epidemiology, and Informatics, Perelman School of Medicine, University of Pennsylvania, Philadelphia; 5 Center for Clinical Epidemiology and Biostatistics, Perelman School of Medicine, University of Pennsylvania, Philadelphia

## Abstract

**Importance:**

Offers of payment for research participation are ubiquitous but may lead prospective participants to deceive about eligibility, jeopardizing study integrity and participant protection. To date, neither the rate of payment-induced deception nor the influence of payment amount has been systematically studied in a nationally representative randomized survey experiment.

**Objectives:**

To estimate payment-associated deception about eligibility for an online survey and to assess whether there is an association between payment amount and deception frequency.

**Design, Setting, and Participants:**

Randomized, 7-group survey experiment. Data were collected in March 2018 and analyzed from March to August 2018. The setting was a nationally representative online survey among US adults drawn from the GfK KnowledgePanel.

**Interventions:**

Varying payment amounts for participation ($5, $10, or $20 cash equivalent) and direction of eligibility criterion (having received or not having received an influenza vaccination in the past 6 months).

**Main Outcomes and Measures:**

Proportion of respondents reporting recent influenza vaccination.

**Results:**

In total, 2275 individuals participated in the survey, a 59.4% (2275 of 3829) response rate; 51.8% (1108) were female, and 21.1% of respondents (399) were aged 18 to 29 years, 24.9% (532) were aged 30 to 44 years, 26.0% (601) were aged 45 to 59 years, and 28.0% (738) were 60 years or older. For participants offered a $5 incentive, the reported frequency of recent influenza vaccination was 16.6% higher (95% CI, 9.1%-24.1%) among those told that eligibility (and thus payment) required recent vaccination than among those told that eligibility required no recent vaccination. The corresponding differences were 21.0% (95% CI, 13.5%-28.5%) among those offered $10 and 15.4% (95% CI, 7.8%-23.0%) among those offered $20. Estimated proportions of ineligible individuals who responded deceptively regarding eligibility ranged from 10.5% to 22.8% across study groups. There was no evidence that higher payment was associated with higher frequency of deception.

**Conclusions and Relevance:**

In a nationally representative randomized survey experiment to evaluate whether and to what extent payment is associated with participants misleading investigators about their research eligibility, this study found evidence of significant deception. However, no association was observed between payment amount and frequency of deception. Further research is needed to extend these findings to clinical research. These data suggest that, when possible, investigators should rely on objective tests of eligibility rather than self-report.

## Key Points

**Question:**

Is payment associated with participant deception about research eligibility and, if so, is higher payment associated with more deception?

**Findings:**

In this randomized survey experiment of a nationally representative sample of 2275 US adults, offers of payment to participate in an online survey were associated with substantial deception by participants about their eligibility compared with the control condition, with estimated proportions of ineligible individuals who engaged in deception ranging from 10.5% to 22.8%. Larger payments were not associated with increased rates of deception.

**Meaning:**

Payment may be associated with deception about eligibility for study participation, but higher payment may not lead to higher rates of deception.

## Teaser

This randomized survey experiment of a nationally representative sample of US adults estimates payment-associated deception about eligibility for an online survey and assesses whether there is an association between payment amount and deception frequency.

## Introduction

Offering payment for research participation is often both practically necessary and ethically fraught. Relying on participant altruism or the prospect of direct medical benefit may be insufficient to achieve recruitment goals, necessitating offers of reimbursement, compensation, and incentives to encourage enrollment and retention.^1^ Concern about undue influence stemming from such payment can be minimized by the requirement that institutional review boards (IRBs) approve research only when its balance of risks and benefits is objectively reasonable.^2^ However, even if ethical concerns can be managed, an important pragmatic concern persists: anecdotal evidence and common sense suggest that payment may encourage participants to mislead investigators about their eligibility or other aspects of participation by concealing or fabricating information to secure payment. Intentional misrepresentation of this nature, otherwise known as deception, may have serious consequences for participant safety and the validity of research results.^3^

Systematic, prospective data regarding whether and to what extent participants will deceive when offered payment in a real study are lacking.^4,5,6,7,8,9^ Therefore, we designed a randomized survey experiment in a nationally representative sample of US adults participating in an online panel, with the objectives of quantifying payment-associated deception about eligibility and evaluating whether variable payment amounts are associated with variable rates of deception.

## Methods

### Design

The University of Pennsylvania IRB approved the study as involving no greater than minimal risk to participants. To avoid influencing participant behavior, the IRB approved an alteration of informed consent to omit a statement disclosing the study’s true objective of evaluating payment-associated deception. Instead, at the end of the survey, each participant was told that “results will be used to help us learn how offering payment affected people’s responses to survey questions. This will help better understand how payment should be used in future research studies.”

Primary data collection for this project was conducted in collaboration with GfK Knowledge Networks, an online survey company. GfK is a member of the American Association for Public Opinion Research (AAPOR) Transparency Initiative and follows all of the association’s reporting guidelines. GfK uses probability-based recruitment procedures to populate its nationally representative survey panel, known as the GfK KnowledgePanel (GfK Custom Research North America), and standing KnowledgePanel members are randomly selected to participate in ongoing studies. Selected members receive an email invitation with a unique password permitting a single use; GfK provides internet access and connected devices to members who lack these tools. The GfK KnowledgePanel allows the generation of fully representative samples to produce statistically valid inferences for study populations.^10^

For this study, GfK invited a random sample to complete a 1-minute online questionnaire about attitudes toward vaccines. The email invitation sent to prospective participants stated the amount of payment offered but not the eligibility criteria, which were disclosed only on clicking the survey link.

Participants were randomly assigned into 7 groups, and the survey was held open from March 2 to 13, 2018, until there were 325 respondents in each group. Control group participants (group 1) were told that they were eligible if they had received any vaccine in their lifetime, a condition intended to achieve near-universal eligibility and avoid any incentive to provide either affirmative or negative answers to a subsequent question specifically about influenza vaccination status (the primary outcome measure). In the 6 experimental groups, participants were exposed to the following 2 interventions: (1) direction of the self-reported eligibility criterion (ie, whether having received an influenza vaccination in the past 6 months made them eligible or ineligible) and (2) amount of incentive. Unlike group 1, these participants were told that they were eligible if they had (A groups) or had not (B groups) received an influenza vaccine in the past 6 months, a period that covered the 2017-2018 influenza season. Eligible participants in groups 2A and 2B were offered a $5 cash equivalent for survey completion, groups 3A and 3B were offered $10, and groups 4A and 4B were offered $20.

Although group 1 participants’ eligibility was not tied to their influenza vaccination status, they were also offered $5 to complete the survey. This was necessary to ensure that the only difference between group 1 participants and groups 2A and 2B participants was whether payment depended on their answer to the question about recent influenza vaccination status; this allowed for isolation of the association between payment and rate of deception regarding self-reported eligibility. Inclusion of groups receiving higher levels of payment (groups 3A and 3B and groups 4A and 4B) allowed testing for dose response according to incentive amount.

In the survey, group 1 participants were first asked if they had ever received a vaccination in their lifetime (question 0 [Q0]); all groups were asked whether they had received an influenza vaccine in the past 6 months (Q1). Because eligibility in group 1 was not dependent on recent influenza vaccination status, group 1 participants had no reason to answer the corresponding question, Q1, deceptively. Accordingly, we used the proportion of group 1 respondents reporting recent influenza vaccination as an estimate of actual baseline prevalence of recent influenza vaccination among the study population (ie, as the control). Inclusion of the A and B groups at each payment amount offered a further internal control; absent deception, reported influenza vaccination rates should not differ between groups 2A and 2B, between groups 3A and 3B, and between groups 4A and 4B.

After Q1, all participants who self-reported eligibility for the survey were presented with 3 statements selected from the Parent Attitudes About Childhood Vaccines (PACV) survey tool.^11^ They were asked to state their level of agreement on a 5-point scale (1 is strongly agree, 2 is agree, 3 is not sure, 4 is disagree, and 5 is strongly disagree) with the following statements: “Children get more shots than are good for them” (Q2), “I believe that many of the illnesses that shots prevent are severe” (Q3), and “It is better for a child to develop immunity by getting sick than to get a shot” (Q4). These statements were included to provide content to the survey so that it would not end immediately after Q1, as well as to test an exploratory hypothesis that deception about eligibility may be associated with biased responses to other survey questions. More specifically, if deception was detected in the A and B groups, we sought to test whether reported vaccine attitudes in these groups would differ from those expressed by similar participants in group 1. The objective was not to assess vaccine attitudes in the study population but rather to evaluate whether participants who were deceptive about their eligibility might also adjust their responses to subsequent survey questions, perhaps based on speculation as to how a truly eligible participant might respond.

GfK provided participants’ demographic data along with survey results. KnowledgePanel members self-report demographics in a multiple-choice format.

### Statistical Analysis

Data were analyzed from March to August 2018. We performed χ^2^ tests within pairs of groups (group 2A v 2B, group 3A v 3B, group 4A v 4B) to compare the proportions of participants who reported influenza vaccination in the past 6 months at each of the 3 payment levels ($5, $10, or $20). Sample sizes (325 respondents per group) were calculated to achieve greater than 90% power to detect a true difference of at least 13 percentage points in reported influenza vaccination rates within each A and B pair (2-sided test, α = .05). We also tested for an association between payment amount and reported influenza vaccination in the A and B groups separately using binary logistic regression.

To estimate the percentage of truly ineligible participants in each experimental group who answered the eligibility question (Q1) deceptively, we assumed that, in the absence of incentives, the distribution of responses in each experimental group would mirror that in the control group. For respondents incentivized to report that they had received an influenza vaccine (A groups), we calculated the percentage of respondents answering deceptively as follows: (% reporting vaccination in the experimental group − % reporting vaccination in the control group) / % reporting no vaccination in the control group. Conversely, for respondents incentivized to report that they had not received an influenza vaccine (B groups), we calculated the percentage of respondents answering deceptively as follows: (% reporting no vaccination in the experimental group − % reporting no vaccination in the control group) / % reporting vaccination in the control group. We assumed that all participants who self-reported ineligibility to participate in the survey were truthful because that response led to no payment. Therefore, they were excluded from these calculations.

To evaluate responses to PACV statements, we developed a single index score for each eligible participant by averaging the responses to the 3 questions after reverse scoring Q3.^12^ Individual index scores were then averaged by group to create a single mean score for those in group 1 reporting recent influenza vaccination, those in group 1 reporting no recent influenza vaccination, those in the A groups reporting recent influenza vaccination (with incentive to do so), and those in the B groups reporting no recent influenza vaccination (with incentive to do so). The mean scores could range from 1 (reflecting maximal vaccine hesitancy) to 5 (reflecting minimal vaccine hesitancy). The mean scores were compared across groups using paired *t* tests.

For this study, design weights were computed to reflect the selection probabilities of assignees. Subsequently, final analysis weights were computed by balancing design weights with respect to geodemographic distributions of the target population for which benchmarks were obtained from the March Supplement 2017 US Census Bureau Current Population Survey.^13^ Respondents were weighted to represent the US adult population 18 years or older. We accounted for final design weights in all analyses. All *P* values are 2-sided, and statistical significance is defined as *P* < .05. Statistical analyses were conducted with a software program (SAS, version 9.4; SAS Institute Inc).

## Results

Among 3829 individuals invited to participate in the survey, 2275 responded, for a response rate of 59.4%. Of the respondents, 51.8% (1108) were female; 21.1% (399) were aged 18 to 29 years, 24.9% (532) were aged 30 to 44 years, 26.0% (601) were aged 45 to 59 years, and 28.0% (738) were 60 years or older. Characteristics of participants, including age, sex, educational level, race/ethnicity, and household income, are listed in Table 1. The study was not designed or powered to assess results by demographic category; demographics are provided solely to demonstrate sample diversity.

**Table 1.** Respondent Demographics by Group^a^

Variable	Group, No. (%)							
	1 (n = 320)^b^	2A (n = 325)^c^	2B (n = 325)^d^	3A (n = 325)^e^	3B (n = 325)^f^	4A (n = 325)^g^	4B (n = 325)^h^	Total (N = 2270)
Age, y								
18-29	63 (21.1)	56 (21.1)	60 (21.1)	57 (21.1)	62 (21.1)	48 (21.1)	53 (21.1)	399 (21.1)
30-44	72 (25.0)	70 (24.9)	78 (24.9)	77 (24.9)	75 (24.9)	85 (24.9)	75 (24.9)	532 (24.9)
45-59	83 (25.7)	86 (26.0)	87 (26.0)	86 (26.0)	85 (26.0)	81 (26.0)	93 (26.0)	601 (26.0)
≥60	102 (28.1)	113 (28.0)	100 (28.0)	105 (28.0)	103 (28.0)	111 (28.0)	104 (28.0)	738 (28.0)
Sex								
Male	160 (48.1)	164 (48.2)	163 (48.2)	169 (48.2)	162 (48.2)	168 (48.2)	176 (48.2)	1162 (48.2)
Female	160 (51.9)	161 (51.8)	162 (51.8)	156 (51.8)	163 (51.8)	157 (51.8)	149 (51.8)	1108 (51.8)
Educational level								
<High school	28 (11.3)	34 (11.1)	25 (11.1)	29 (11.1)	34 (11.1)	30 (11.1)	28 (11.1)	208 (11.1)
High school	89 (28.7)	81 (28.9)	84 (28.9)	84 (28.9)	89 (28.9)	89 (28.9)	91 (28.9)	607 (28.9)
Some college	96 (28.7)	92 (28.6)	96 (28.6)	101 (28.6)	97 (28.6)	95 (28.6)	105 (28.6)	682 (28.6)
≥Bachelor’s degree	107 (31.3)	118 (31.4)	120 (31.4)	111 (31.4)	105 (31.4)	111 (31.4)	101 (31.4)	773 (31.4)
Race/ethnicity								
White, non-Hispanic	233 (64.5)	228 (64.0)	231 (64.0)	234 (64.0)	224 (64.0)	231 (64.0)	225 (64.0)	1606 (64.1)
Black, non-Hispanic	28 (11.7)	25 (11.8)	27 (11.8)	28 (11.8)	28 (11.8)	26 (11.8)	36 (11.8)	198 (11.8)
Other, non-Hispanic	8 (6.5)	16 (7.0)	14 (6.8)	14 (7.4)	9 (7.3)	15 (6.9)	13 (6.9)	89 (7.0)
Hispanic	43 (15.4)	46 (15.9)	42 (15.9)	41 (15.9)	57 (15.9)	41 (15.9)	41 (15.9)	311 (15.8)
>1 Race/ethnicity, non-Hispanic	8 (1.9)	10 (1.2)	11 (1.4)	8 (0.8)	7 (1.0)	12 (1.4)	10 (1.4)	66 (1.3)
Annual household income, $								
<25 000	48 (15.3)	49 (15.1)	41 (15.1)	52 (15.1)	72 (15.1)	49 (15.1)	42 (15.1)	353 (15.1)
25 000-49 999	64 (19.7)	68 (19.8)	70 (19.8)	56 (19.8)	57 (19.8)	68 (19.8)	72 (19.8)	455 (19.8)
50 000-74 999	62 (16.9)	52 (17.3)	53 (17.3)	56 (17.3)	61 (17.3)	51 (17.3)	58 (17.3)	393 (17.3)
75 000-99 999	45 (14.1)	46 (13.8)	48 (13.8)	50 (13.8)	39 (13.8)	53 (13.8)	53 (13.8)	334 (13.9)
100 000-149 999	52 (17.0)	54 (17.0)	56 (17.0)	63 (17.0)	49 (17.0)	61 (17.0)	56 (17.0)	391 (17.0)
≥150 000	49 (16.9)	56 (17.0)	57 (17.0)	48 (17.0)	47 (17.0)	43 (17.0)	44 (17.0)	344 (17.0)

^a^ The numbers in each cell are the unweighted sample size and the weighted column percentage. Design weights were computed to reflect the selection probabilities of assignees, as described in the Design subsection of the Methods section.

^b^ Eligibility criterion: any vaccine in lifetime. Payment amount: $5. An additional 5 respondents in group 1 were considered ineligible because they reported never receiving a vaccine in their lifetimes.

^c^ Eligibility criterion: influenza vaccine in the past 6 months. Payment amount: $5.

^d^ Eligibility criterion: no influenza vaccine in the past 6 months. Payment amount: $5.

^e^ Eligibility criterion: influenza vaccine in the past 6 months. Payment amount: $10.

^f^ Eligibility criterion: no influenza vaccine in the past 6 months. Payment amount: $10.

^g^ Eligibility criterion: influenza vaccine in the past 6 months. Payment amount: $20.

^h^ Eligibility criterion: no influenza vaccine in the past 6 months. Payment amount: $20.

In the control group, 52.2% (weighted 95% CI, 46.3%-58.1%) of respondents (173 respondents unweighted) who reported ever having received any vaccine reported having received an influenza vaccine in the past 6 months (“recent vaccination”). Absent deception, this rate should have remained constant across all study groups. However, it increased to 63.1% (95% CI, 57.4%-68.7%) in group 2A, 62.8% (95% CI, 57.3%-68.4%) in group 3A, and 62.1% (95% CI, 56.5%-67.8%) in group 4A, all of which were incentivized to self-report recent influenza vaccination (Table 2). Conversely, it decreased to 46.5% (95% CI, 40.5%-52.4%) in group 2B, 41.8% (95% CI, 36.0%-47.5%) in group 3B, and 46.7% (95% CI, 40.9%-52.5%) in group 4B, all of which were incentivized to self-report no recent influenza vaccination (*P* < .001 for the 7-way comparison). This reflects differences of 16.6% (95% CI, 9.1%-24.1%) in reported recent vaccination between groups 2A and 2B, 21.0% (95% CI, 13.5%-28.5%) between groups 3A and 3B, and 15.4% (95% CI, 7.8%-23.0%) between groups 4A and 4B (*P* < .001 for all 2-way comparisons).

**Table 2.** Responses to the Eligibility Question by Payment Amount

Group	Eligibility Condition^a^	Payment Amount, $	Unweighted No. Reporting Recent Influenza Vaccine	Weighted % Reporting Recent Influenza Vaccine (95% CI)	Difference Between A Groups and B Groups, % (95% CI)	*P* Value	Estimated % of Truly Ineligible Participants Who Answered Deceptively
1 (n = 320)^b^	Neutral (any vaccine ever)	5	173	52.2 (46.3-58.1)	NA		NA
2A (n = 325)	Recent influenza vaccine	5	208	63.1 (57.4-68.7)	16.6 (9.1-24.1)	<.001	22.8
2B (n = 325)	No recent influenza vaccine	5	159	46.5 (40.5-52.4)			10.9
3A (n = 325)	Recent influenza vaccine	10	203	62.8 (57.3-68.4)	21.0 (13.5-28.5)	<.001	22.2
3B (n = 325)	No recent influenza vaccine	10	138	41.8 (36.0-47.5)			19.9
4A (n = 325)	Recent influenza vaccine	20	203	62.1 (56.5-67.8)	15.4 (7.8-23.0)	<.001	20.7
4B (n = 325)	No recent influenza vaccine	20	151	46.7 (40.9-52.5)			10.5

Abbreviation: NA, not applicable.

^a^ Recent refers to influenza vaccine in the past 6 months.

^b^ An additional 5 respondents in group 1 were considered ineligible because they reported never receiving a vaccine in their lifetimes. The study groups are fully described in the Design subsection of the Methods section.

Based on the estimated true proportion of recent influenza vaccination in the study population (52.2%), estimated proportions of participants who were actually not eligible to participate but who answered deceptively about their eligibility ranged from 10.5% to 22.8% in the 6 experimental groups (Table 2). There was no evidence of a dose-response relationship with increasing payment amounts either among participants incentivized to report or not report recent influenza vaccination. In both the A and B groups, self-reported influenza vaccination rates remained almost flat across increasing incentives to deceive, as noted earlier (Figure).

**Figure.**
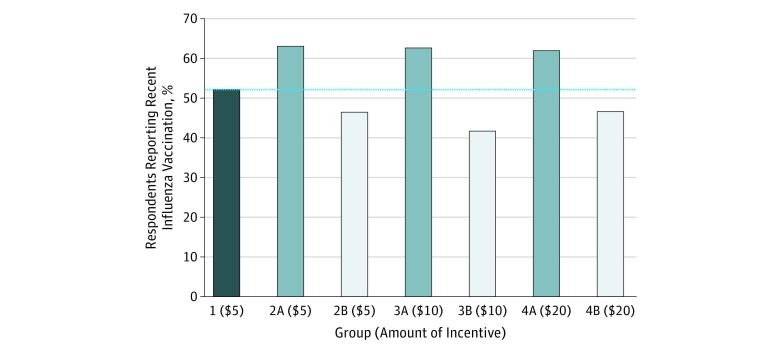
Percentage of Respondents Reporting Influenza Vaccination in the Previous 6 Months Bars show the percentages of respondents in each group who self-reported that they had received an influenza vaccine in the previous 6 months. Respondents in group 1 had no financial incentive to respond affirmatively or negatively to the question about recent influenza vaccination; the dashed line indicates the group 1 rate of reported recent influenza vaccination, which should be the same across all study groups in the absence of deception about eligibility in A and B groups. Respondents in groups 2A, 3A, and 4A had a financial incentive to respond that they had received a recent influenza vaccine. Respondents in groups 2B, 3B, and 4B had a financial incentive to respond that they had not received a recent influenza vaccine. *P* values for the 7-way comparison and for all 2-way comparisons between A groups and B groups were less than .001, indicating statistically significant levels of deception in the intervention groups. In contrast, the *P* value for the 3-way comparison across the A groups was .81 and across the B groups was .75, providing no statistically significant evidence of dose response based on increasing payment amount.

The mean score on PACV questions among group 1 (control) participants reporting recent influenza vaccination was 3.87 (95% CI, 3.75-4.00) of 5; among group 1 participants reporting no recent influenza vaccination, it was 3.58 (95% CI, 3.44-3.72). Eligible participants in the A groups (reporting recent influenza vaccination) had a combined mean score of 3.81 (95% CI, 3.74-3.87), and eligible participants in the B groups (reporting no recent influenza vaccination) had a combined mean score of 3.43 (95% CI, 3.36-3.50). We observed no significant difference in the mean scores between group 1 respondents reporting recent influenza vaccination and those reporting eligibility in the A groups (3.87 vs 3.81, *P* = .35) or between group 1 respondents reporting no recent influenza vaccination and those reporting eligibility in the B groups (3.58 vs 3.43, *P* = .06) (Table 3).

**Table 3.** Vaccine Attitude Scores

Reported Recent Influenza Vaccine	Presence and Direction of Incentive	No. of Respondents	Mean Score (95% CI)^a^	*P* Value
Yes	No incentive to report recent influenza vaccine^b^	172	3.87 (3.75-4.00)	.35
	Incentive to report recent influenza vaccine^c^	612	3.81 (3.74-3.87)	
No	No incentive to report recent influenza vaccine^b^	146	3.58 (3.44-3.72)	.06
	Incentive to report no recent influenza vaccine^d^	526	3.43 (3.36-3.50)	

^a^ Score range of 1 (maximal vaccine hesitancy) to 5 (minimal vaccine hesitancy).

^b^ Subset of group 1. The study groups are described in the Design subsection of the Methods section.

^c^ Groups 2A, 3A, and 4A combined.

^d^ Groups 2B, 3B, and 4B combined.

## Discussion

In this randomized survey experiment conducted in a nationally representative sample of US adults, we observed evidence of substantial payment-associated deception regarding self-reported eligibility even at the lowest amount of payment offered. However, we did not observe evidence that higher payment was associated with more frequent deception. These findings provide novel insight regarding the potential association between offers of payment and deception by participants.

Our study was motivated by gaps in the empirical literature on payment-induced deception. Prior studies have raised concern that payment may lead research participants to mislead investigators, but the studies are limited to identifying cases in the published literature, retrospective self-report, hypothetical projections of potential behavior, and nonrepresentative populations. For example, Lee et al^3^ identified 103 instances of deception, including concealment, fabrication, drug holidays, and collusion, in 90 studies selected from a literature review of articles that included the terms *deception*, *deceit*, and *subversive subjects*, among others. Most of these instances of deception were incidentally detected, and the authors of the review article acknowledge that “few studies have investigated deceit in research participants, and fewer studies have examined deceit as a primary objective.”^3^^(p154)^ Our study did both.

Walker et al^4^ reported evidence of “rule-breaking” with implications for study validity and participant safety, much of which was motivated by payment, based on 5 case studies of repeat participants in phase 1 clinical trials drawn from a larger set of interviews with healthy volunteers. Relying on the same set of interviews with healthy volunteers (more than two-thirds of whom had participated in ≥5 clinical trials), McManus and Fisher^5^ found that payment can influence reporting of adverse events, but participants may be willing “to forgo their full compensation if they believe not reporting their symptoms jeopardizes their own safety or the validity of the research.”^5^^(p82)^ In a survey of individuals who had participated in at least 2 studies in the past year (of any type but most frequently “medication trials”), Devine et al^6^ found high rates of self-reported lifetime deceptive behavior related to eligibility for clinical trials, with significant correlation between deception and monetary reward.

Other studies have evaluated the hypothetical influence of payment on deception. Bentley and Thacker^7^ asked US pharmacy students to rate willingness to participate in hypothetical clinical studies and the likelihood they would engage in various disclosures to investigators. They found that higher levels of payment may influence behaviors regarding concealing information about restricted activities, but payment did not have a significant association with the likelihood of reporting adverse events. Slomka et al^8^ interviewed “economically disadvantaged African-American crack cocaine smokers”^8^^(p1403)^ participating in HIV prevention studies to solicit their general views about payment for research participation and identified some concern that high payments might cause people to provide false information to investigators. Finally, outside of the clinical trial context, Chandler and Paolacci^9^ carried out a series of online experiments using Amazon’s Mechanical Turk platform and determined that participants often misrepresent relevant characteristics to meet the eligibility criteria.

Our study builds on these findings by analyzing deception in a broader population sample offered payment to participate. The randomized nature of the experiment allowed us to assess the association of payment, payment amount, and direction of the incentive with aggregate deception about self-reported eligibility to participate. Although this study was carried out in the context of survey research, the data offer a rigorous and internally valid signal about the potential influence of payment on deception by research participants. Our main finding that there was significant payment-associated deception is broadly relevant to the work of all scientists who carry out data collection with human participants, including clinical trialists using survey methods to evaluate participant-reported outcomes or otherwise supplement clinical data, as well as those conducting trials of interventions relying on self-reported adherence, because these data may be influenced by participant desire to continue receiving study payment.^14,15^ Our results offer a compelling reason to carry out similar research in the clinical trial context, where payment amounts and other benefits—such as access to potentially desirable investigational medicines—are often more substantial than typically associated with survey research. They also suggest that trialists should use objective rather than self-reported eligibility (and other) criteria whenever possible and that deception regarding eligibility may be a particular concern for pragmatic trials enrolling participants on the basis of self-reported behaviors and conditions.

The absence of higher rates of deception at higher payment amounts in our study may reflect that some proportion of individuals will not deceive even when the financial reward of doing so is great or that some individuals have “tipping points” higher than the amounts we offered. Addressing these questions about dose response is important because it may be that attempts to avoid deception by keeping offers of payment low will be ineffective (based on our finding that deception may occur even at low payment amounts) and that high payment offers may have the benefit of speeding recruitment without posing greater risk to study integrity (based on our finding that deception may not increase at higher payment amounts). In either case, as noted in the previous paragraph, adopting objective eligibility criteria should be a goal whenever possible.

A potential result of deception about eligibility is that the validity of other information reported by participants may also be affected. We hypothesized that individuals who deceived about eligibility might seek to answer subsequent questions regarding vaccine attitudes in a biased manner according to their estimate of how someone who was truly eligible might answer. However, it is unclear whether participants engaged in such role playing. Based on the absence of statistical significance on comparative mean vaccine attitude scores, we found no evidence that deception about eligibility by participants who were incentivized to report having either received or not received a recent influenza vaccine influenced responses to other survey questions.

### Limitations and Research Agenda

The primary limitation of our study is that it was conducted in the context of a survey relying on self-reported eligibility; therefore, it cannot directly answer questions about the possibility and influence of payment-induced deception in the clinical trial context. In addition, there was no comparison of responders (59.4%) with nonresponders, which may limit generalizability of these findings. In contrast to some clinical trials that may entail high levels of burden and risk, participants in this brief online survey were asked to undertake little burden and no risk for proportionately high payment. They could also engage in deception without fear of being caught or of incurring physical risk by evading eligibility criteria intended to protect them. It is unclear whether clinical trial participants will be more inclined to deceive as a result of typically higher offers of payment in that setting or less inclined given greater potential consequences for themselves or investment in the outcome of the research.

Our study design cannot rule out the possibility that reported rates of recent influenza vaccination varied according to the eligibility criteria not as a result of payment-associated deception but rather as a result of participant desire to report eligibility in a way that would assist researchers (a social desirability or “helpfulness” bias). However, there is no evidence to suggest that individuals are eager to participate in research for which they are not eligible in the absence of incentive payments.

Another limitation is that we tested only 3 payment levels, which could obscure signals that may have become evident by evaluating more payment amounts at tighter increments. In addition, our assessment of whether payment was associated with different information provided by research participants after deception about eligibility was limited to an exploratory measure based on a small number of additional questions regarding vaccine attitudes not validated for that particular purpose.

One pressing research need is to replicate these findings among participants in clinical trials that have some self-reported criteria for initial and continuous eligibility. Further research is also needed to understand whether a dose-response relationship between payment amount and deception rates might be seen in other contexts or at different amounts, as well as to systematically assess the downstream influence of the inclusion of individuals in research who were deceptive about their eligibility. As further data are developed, research evaluating the effectiveness of approaches to minimize payment-induced deception will also be important.

## Conclusions

Ethical concerns about undue influence have dominated discussions about payment for research participation, but investigators and IRBs also should be aware of the potential for payment to interfere with a study’s scientific integrity and participant protections. Our study found that as many as 10.5% to 22.8% of ineligible participants were motivated to deceive about their eligibility to enroll in an online survey to secure payment, although higher rates of payment were not associated with higher rates of deception. Because offering payment can be an important and ethical tool for recruiting, retaining, and fairly compensating participants,^1^ we caution against responding to these findings by eliminating or reducing offers of payment. Instead, it is necessary to develop, test, and implement comprehensive alternative approaches to minimize the likelihood of participant deception.^3^

## References

[1] Gelinas L, Largent EA, Cohen IG, Kornetsky S, Bierer BE, Fernandez Lynch H A framework for ethical payment to research participants. N Engl J Med. 2018;378(8):-. doi:10.1056/NEJMsb1710591 29466147

[2] Largent EA, Lynch HF Paying research participants: the outsized influence of “undue influence.” IRB. 2017;39(4):1-9. 29038611 PMC5640154

[3] Lee CP, Holmes T, Neri E, Kushida CA Deception in clinical trials and its impact on recruitment and adherence of study participants. Contemp Clin Trials. 2018;72:146-157. doi:10.1016/j.cct.2018.08.002 30138717 PMC6203693

[4] Walker RL, Cottingham MD, Fisher JA Serial participation and the ethics of phase 1 healthy volunteer research. J Med Philos. 2018;43(1):83-114. doi:10.1093/jmp/jhx033 29342285 PMC5901090

[5] McManus L, Fisher JA To report or not to report: exploring healthy volunteers’ rationales for disclosing adverse events in phase I drug trials. AJOB Empir Bioeth. 2018;9(2):82-90. doi:10.1080/23294515.2018.1469552 29693508 PMC5976538

[6] Devine EG, Waters ME, Putnam M, Concealment and fabrication by experienced research subjects. Clin Trials. 2013;10(6):935-948. doi:10.1177/1740774513492917 23867223

[7] Bentley JP, Thacker PG The influence of risk and monetary payment on the research participation decision making process. J Med Ethics. 2004;30(3):293-298. doi:10.1136/jme.2002.001594 15173366 PMC1733848

[8] Slomka J, McCurdy S, Ratliff EA, Timpson S, Williams ML Perceptions of financial payment for research participation among African-American drug users in HIV studies. J Gen Intern Med. 2007;22(10):1403-1409. doi:10.1007/s11606-007-0319-9 17668270 PMC2305851

[9] Chandler JJ, Paolacci G Lie for a dime: when most prescreening responses are honest but most study participants are impostors. Soc Psychol Personal Sci. 2017;8(5):500-508. doi:10.1177/1948550617698203

[10] KnowledgePanel. Recruitment and sample survey methodologies. https://www.gfk.com/fileadmin/user_upload/dyna_content/US/documents/KnowledgePanel_Methodology.pdf. Accessed July 3, 2018.

[11] Opel DJ, Mangione-Smith R, Taylor JA, Development of a survey to identify vaccine-hesitant parents: the Parent Attitudes About Childhood Vaccines survey. Hum Vaccin. 2011;7(4):419-425. doi:10.4161/hv.7.4.14120 21389777 PMC3360071

[12] Opel DJ, Taylor JA, Mangione-Smith R, Validity and reliability of a survey to identify vaccine-hesitant parents. Vaccine. 2011;29(38):6598-6605. doi:10.1016/j.vaccine.2011.06.115 21763384

[13] US Census Bureau Current Population Survey (CPS). https://www.census.gov/programs-surveys/cps/about.html. March Supplement 2017. Accessed August 8, 2018.

[14] Montgomery ET, Mensch B, Musara P, Misreporting of product adherence in the MTN-003/VOICE trial for HIV prevention in Africa: participants’ explanations for dishonesty. AIDS Behav. 2017;21(2):481-491. doi:10.1007/s10461-016-1609-1 27858268 PMC5290166

[15] Stadler J, Scorgie F, van der Straten A, Saethre E Adherence and the lie in a HIV prevention clinical trial. Med Anthropol. 2016;35(6):503-516. doi:10.1080/01459740.2015.1116528 26575611 PMC4977196

